# Quiescence of postharvest pathogens: a fungal inhibition process or an immune response of the unripe host fruit?

**DOI:** 10.1111/nph.70913

**Published:** 2026-02-01

**Authors:** Dov B. Prusky, Tong Chen, Yong Chen, Shiping Tian, Boqiang Li

**Affiliations:** ^1^ Department of Postharvest Science of Fresh Produce, Agricultural Research Organization The Volcani Center Rishon LeZion 7505101 Israel; ^2^ State Key Laboratory of Plant Diversity and Specialty Crops, Institute of Botany Chinese Academy of Sciences Beijing 100093 China; ^3^ China National Botanical Garden Beijing 100093 China; ^4^ University of Chinese Academy of Sciences Beijing 100049 China

**Keywords:** disease resistance, fruit quality, pathogenicity, postharvest diseases, preharvest diseases, storage

## Abstract

Postharvest pathogens can infect fresh produce both before and after harvest, by direct or wound‐enhanced penetration, remaining quiescent until ripening. Biotrophic‐like postharvest pathogens persist beneath host cells and can remain in a state of quiescence. They detect environmental cues and regulate quiescence through chromatin‐level control and the secretion of effectors that interact with host pattern recognition receptors. By contrast, necrotrophic fungi persist between dead cells and depend more directly on nutrient availability to prime their growth and upon secretion for fungal virulence factors. During quiescence, the host also mounts specific responses, including activation of pattern recognition receptor genes, ethylene signaling (particularly in unripe fruit), and defense genes such as *PR‐10* and chitinases. Jasmonic acid and ethylene pathways synergistically enhance these defenses. As fruit ripens, the transition from quiescence to active necrotrophic growth is triggered, accelerating tissue decay. This activation is driven by several key factors, including weakened host defenses, decreased levels of antifungal compounds such as polyphenols, increased cell wall accessibility due to fruit softening and ripening‐associated changes in signaling pathways, which alter environmental pH, carbon metabolism, and secondary metabolite production. These regulatory mechanisms collectively govern the timing and extent of fungal initiation of colonization during fruit senescence.

## Introduction

Fruits and vegetables are highly perishable commodities, and improper handling during harvesting, transportation, or storage can result in significant postharvest losses and waste. The Food and Agriculture Organization (FAO) of the United Nations estimates that about one‐third of global food production – *c*. 1.3 billion metric tons – is lost or wasted each year (Sagar *et al*., 2018). In the case of horticultural products, losses can reach up to 60%, occurring at various stages of the supply chain, from harvest to household consumption (Prusky & Romanazzi, 2023).

Postharvest fungal infections may be initiated before, during, or after harvest but often remain dormant in a quiescent stage until fruit ripening and senescence (Prusky, 1996; Prusky *et al*., 2013). This is different from fungal pathogens that show a complete full cycle in living tissue; for example, Ustilago (Yu *et al*., 2023), which are not covered here. Disease prevention is typically achieved through a combination of fungicide treatments and optimized storage conditions (Adaskaveg *et al*., 2023). Symptoms usually become visible only after prolonged cold storage or during shelf life, just before consumption. Notably, while most freshly harvested fruits and vegetables harbor quiescent infections, the mechanisms governing fungal quiescence and host resistance in unripe fruits remain poorly understood (Adaskaveg *et al*., 2000; Prusky *et al*., 2013). Although recent advances have introduced stable and robust sensing materials with high sensitivity for detecting fruit infections by pathogens, no practical applications have been implemented to date (Archana *et al*., 2024). This knowledge gap underscores the importance of investigating the factors that regulate quiescence and its activation, as such insights could inform the development of novel disease management strategies aimed at reducing reliance on postharvest fungicides while preserving fruit quality over time (Prusky *et al*., 2013).

## What are the infection mechanisms of postharvest pathogens that become quiescent

Postharvest pathogens infect fruits and vegetables through two main mechanisms: direct penetration of the cuticle or entry through wounds, both of which can occur at pre‐ and postharvest stages. Fungal host specificity refers to a pathogen's ability to infect only particular host species, ranging from narrow specificity – such as *Penicillium digitatum* infecting only citrus fruit – to broad specificity, as seen with *Botrytis cinerea*, which infects a wide range of fruit and vegetable hosts (Adaskaveg *et al*., 2023). When a host‐specific postharvest pathogen attempts to infect a nonhost species, the expected outcome is pathogen death and effective host immunity. Host specificity is governed by a complex interplay of genetic, structural, and environmental factors, including the pathogen's capacity to overcome host defenses and the host's inherent genetic resistance mechanisms (Jones & Dangl, 2006; J. Li *et al*., 2020). In postharvest fruit and vegetable crops, these interactions become even more intricate due to the variable responses of the same host – ranging from immune to susceptible – across different stages of development and ripening (Prusky & Romanazzi, 2023), highlighting the complexity of host–pathogen dynamics.

Early in fruit development, host resistance prevents pathogen colonization and forces the microorganisms into quiescence, where no external symptoms are observed. As the fruit ripens and senesces, host defenses weaken and susceptibility increases, allowing pathogen colonization to proceed (Cantu *et al*., 2008a,b). These quiescent infections manifest in two distinct patterns: (1) localized penetrating infection (LPI), where fungi breach specific fruit tissues at various developmental stages (Prusky, 1996); and (2) nonlocalized symptomless infections (NLSI), characterized by endophytic spread through vascular tissues, ultimately attacking the fruit at a distance from the initial infection site. This manuscript focuses on well‐characterized aspects of LPI, while the NLSI type of infection will not be covered in this review.

The LPI can be established in multiple locations of plant tissues, such as flower petals, receptacles, stamens, calyxes, fruit stems, and cuticles (Prusky, 1996). These infections develop through either direct cuticle penetration or wound entry during host growth and handling until fruit maturation triggers symptom development. Numerous fungal species, including *Colletotrichum*, *Alternaria*, *Botrytis*, *Monilinia*, *Lasiodiplodia*, and *Phomopsis*, have been documented to exist in this quiescent state within their hosts (Adaskaveg *et al*., 2000; Prins *et al*., 2000; Prusky *et al*., 2013).

**Fig. 1:**
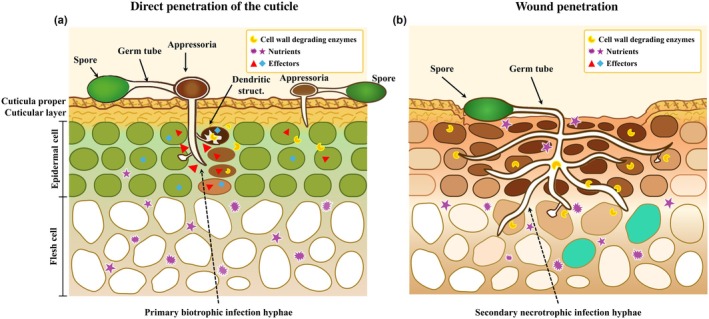
*Colletotrichum* and *Monilinia* establish quiescent infections in fruit either through (a) direct penetration or (b) minor wounds, using hyphae originating from germinating spores. Colored appressoria indicate melanization of the structure. Darker staining of host cells signifies cells in the process of dying. Germinated hyphae remain dormant beneath the cuticle or penetrate one to two cell layers, penetrating to cells using their biotrophic capability and further forming dendritic structures before the cells become necrotic. The limited number of dead cells at each infection site prevents the formation of visible necrotic lesions during quiescent infection. Wound penetration occurs without appressorium formation. The accumulation of reactive oxygen species (ROS) in wounded tissue causes molecular damage, ultimately leading to cell death and facilitating initial fungal colonization (Wang *et al*., 2019). Following this process, the direct biotrophic penetration of cells occurs. *Colletotrichum* will secrete novel cell death‐inducing effector proteins (Takahara *et al*., 2021; Wang *et al*., 2024), but they are mainly produced when the fungus is released from quiescence.

Biotrophic pathogens such as *Colletotrichum* and *Monilinia* establish localized infections by penetrating the cuticle or taking advantage of minor wounds in cuticle and significant stem end wounds (Fig. 1). These pathogens remain suppressed within the host cuticle or beneath the first layer of host cells until fruit ripening compromises the plant's resistance. Necrotrophs such as *B. cinerea* may penetrate flower stigmatic fluid before asymptomatic spread, whereas in kiwifruit, it infects mainly through the stem end wounds (Adaskaveg *et al*., 2023). Both biotrophic and necrotrophic pathogens show an early stage of quiescence during the unripe stage of fruit development (Prusky & Lichter, 2007; Petrasch *et al*., 2019).

Several forms of quiescence have been identified: (1) delayed germination of conidia or postgermination growth arrest of appressoria; (2) asymptomatic endophytic growth in the apoplast; and (3) colonization of abscising floral organs, such as petals, followed by growth arrest in the ovaries or receptacles (Petrasch *et al*., 2019). Ripening‐induced susceptibility triggers pathogen activation, underscoring the importance of understanding quiescence mechanisms for effective postharvest disease control (Prusky, 1996; Ploetz, 2003; Galsurker *et al*., 2020). Infection structures are key factors for either attaching and breaching the intact cuticle or the wounded tissue for fungal infection. Spores that germinated on the cuticle surface can form globular or lobate, melanized, or nonmelanized appressoria (Podila *et al*., 1993; Lee & Bostock, 2006; Oliveira Lino *et al*., 2020). In avocado, the long‐chain fatty alcohols (≥ C24) present in the avocado epicuticular wax critically stimulate *Colletotrichum* appressoria formation, whereas nonhost waxes suppress it (Podila *et al*., 1993; Prusky, 1996). Upon germination, a needle‐like hypha emerges from the base of the appressorium and punctures the cuticle. After appressoria differentiation from germ tubes and penetration, the structure may either remain dormant within the cuticle – as observed in avocado (Daykin, 1984; Prusky, 1996) – or further develop into dendritic structures in the first cell layer beneath the cuticle, as seen in tomato (Alkan *et al*., 2015) (Fig. 1a). Early infection typically avoids necrosis or visible host reactions, likely due to fungal effectors suppressing plant defenses (Kleemann *et al*., 2012; Giraldo & Valent, 2013); however, this may vary according to the host fruit. Following its formation, the germinated appressoria in *Colletotrichum*, *Monilinia*, and *Alternaria* breach the cuticle through a tightly regulated process involving DNA replication, GTPase‐mediated cell cycle progression (G1 to S phase), and melanin deposition (Wao & Köller, 1994; Fan & Köller, 1998; Fetzner *et al*., 2014; Alkan *et al*., 2015; Tang *et al*., 2017; Li *et al*., 2022).

In *B. cinerea*, quiescent infections can occur without appressoria or with either unicellular or multicellular infection cushions (ICs). These nonmelanized structures penetrate host tissues within 24–48 h of spore germination across various plant organs (Adaskaveg *et al*., 2000; Haile *et al*., 2019, 2020; Petrasch *et al*., 2019; Choquer *et al*., 2021; Bi *et al*., 2023). Most *Botrytis* infections in strawberries begin when spores germinate on flower organs (primary infections) during or right after flowering, allowing hyphae to grow into the receptacle (Bristow *et al*., 1986). Infected senescent petals, stamens, and calyxes can facilitate primary infections in fruit (Petrasch *et al*., 2019). In other cases, the stigmatic fluid provides nutrients for airborne conidia, facilitating their growth in the humid microclimate in the raspberry (Williamson *et al*., 2007). The infection then spreads to the receptacle before becoming arrested and entering a quiescent state (Bristow *et al*., 1986; Jarvis, 1994; Prusky, 1996). In grapes, a direct germination and infection of *Botrytis* was linked to cuticle thickness, which restricts water and nutrient diffusion through water permeability sites (Herzog *et al*., 2015). While cultivars with thinner hydrophobic wax layers promote surface water and nutrient spread, increasing infection rates.

**Fig. 2:**
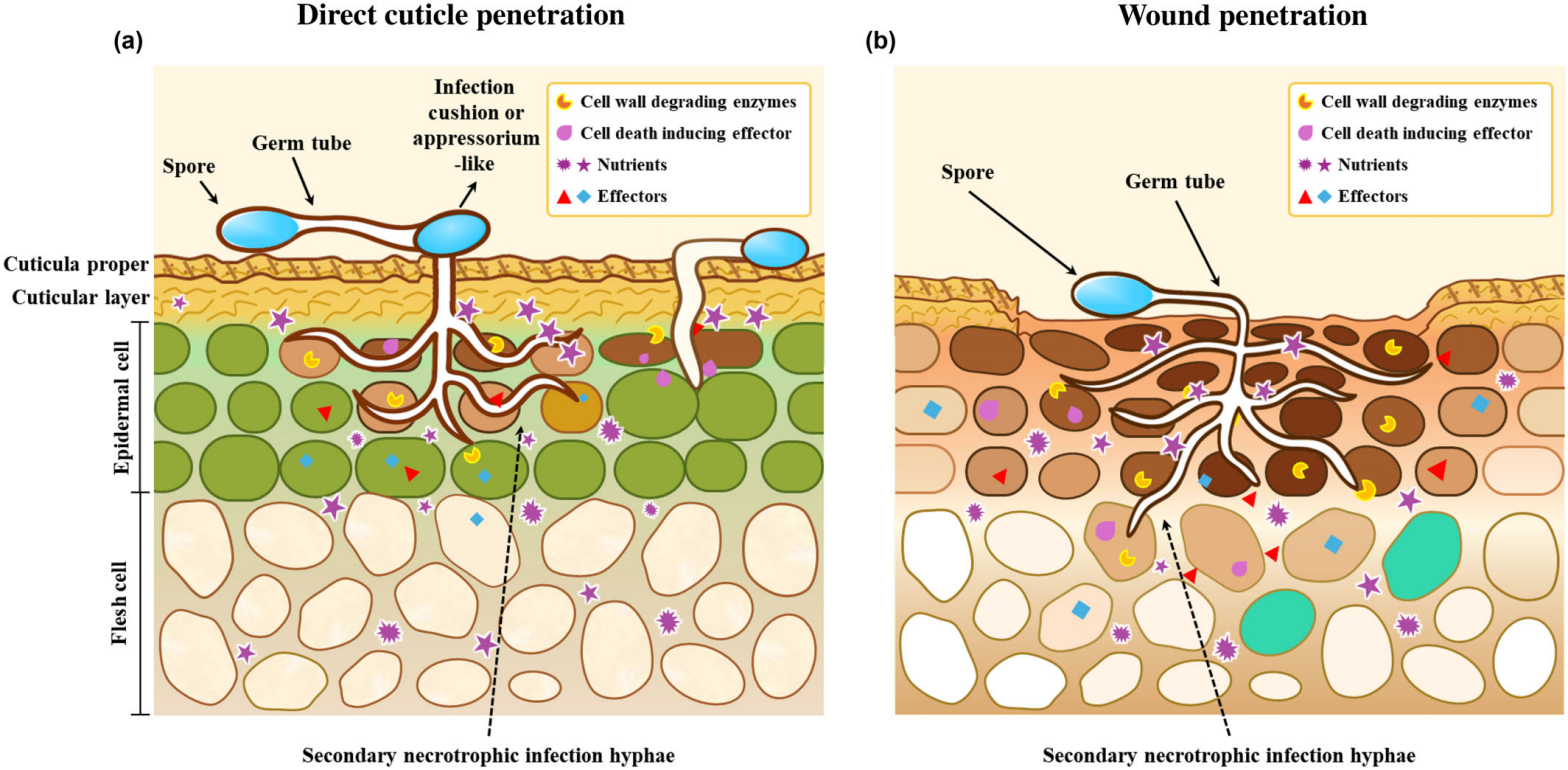
*Botrytis* establishes quiescent infections in fruit either through (a) direct penetration or (b) minor wounds, using hyphae originating from germinating spores. Darker staining of host cells signifies cells in the process of dying. Germinated spores produce either an infection cushion or a nonmelanized appressoria‐like structure that develops a penetrating hypha that remains dormant beneath the cuticle after penetration of one to two cell layers between dead cells. A very short biotrophic stage that lasts *c*. 1 d up to the entrance into necrotrophy is observed (Shlezinger *et al*., 2011; Veloso & van Kan, 2018; Bi *et al*., 2023). This is compared to a more extended (many days) biotrophic stage of fungi as *Colletotrichum* and *Monilinia*, that display true appressoria and a distinct biotrophic stage (Alkan *et al*., 2015). The brief biotrophic phase, during which autophagy is suppressed before the induction of apoptotic cell death, marks the onset of necrotic disease development and suggests a required preliminary step in the necrotization process (Veloso & van Kan, 2018). (b) Wound penetration occurs without the formation of an infection cushion or appressoria‐like structures. The accumulation of reactive oxygen species (ROS) in wounded tissue causes cell death, and the nutritional availability facilitates the initial secretion of cell death‐inducing proteins and fungal colonization (Lin *et al*., 2025), conditions where the fungus remains quiescent.

In other described cases of *Botrytis*, spores may form appressoria‐like structures, from which hyphae can emerge at nearly perpendicular angles (Fig. 2a) (Bi *et al*., 2023). Penetration is driven by turgor pressure, supported by the actin cytoskeleton, and is accompanied by the secretion of effectors, including phytotoxins, proteases, and cell wall–degrading enzymes. Unlike melanized appressoria in other fungi, *B. cinerea* depends more on enzymatic degradation than mechanical force for penetration. Early infection triggers grapevine defenses, including antimicrobial proteins and cell wall reinforcement, which suppress fungal growth until favorable conditions reactivate pathogenesis (Haile *et al*., 2019).

In summary, postharvest pathogens first detect the host and then regulate diverse metabolic processes to: (1) develop penetration structures; (2) identify optimal entry sites; (3) equip these structures with cutinase and lipases to breach the host cuticle, ensuring successful quiescence; and (4) inhibit fungal colonization and host immunity until fruit ripening. Therefore, understanding this host–pathogen mechanism during quiescence and its activation proves critical for the prevention of fruit decay throughout postharvest storage life (Bruton *et al*., 1998; Prusky *et al*., 2010).

## The dynamics of the quiescent infection

While numerous studies have documented the initial interactions between postharvest pathogens and their plant hosts (*Colletotrichum* and *Botrytis*) (Tian *et al*., 2016), a limited amount of data exists regarding the fungus's quiescence interactions with fruit. In leaves, the first line of defense against *Botrytis* involves pathogen‐associated molecular pattern (PAMP)‐triggered immunity (PTI), where plant cell surface pattern recognition receptors (PRRs) detect microbial PAMPs and activate basal resistance responses. The second layer, effector‐triggered immunity (ETI), relies on intracellular nucleotide‐binding leucine‐rich repeat receptors (NLRs) that recognize pathogen effectors, triggering a robust immune response often accompanied by localized cell death to restrict pathogen growth (Jones & Dangl, 2006; Ngou *et al*., 2022). Notably, in *postharvest pathogens*, most immune responses linked to quiescence in fruits appear to involve a combination of the first defense layer, with complex deposition between the cuticle and plant cell wall of inducible preformed antifungal compounds (Prusky & Keen, 1993) as well as lignin biosynthesis (Li *et al*., 2025), and also the second layer of defense, where a germinated appressorium breaches the infected host which involves a hypersensitive response (HR) cell death and a systemic activation of plant defenses from the site of signal perception (Alkan *et al*., 2015; Li *et al*., 2025). The initial interaction is characterized by limited intercellular space formation and the penetration of one to two cells, with no visible signs of necrosis (Guidarelli *et al*., 2011).

Recently published data raised several critical questions concerning the phenomenon of quiescence, suggesting that fruit immunity during quiescence may be the result of individual immunity processes or a synergistic collaboration between PTI and ETI to bolster disease resistance and collectively trigger a cascade of downstream defense responses. This will be analyzed in the following paragraphs where the mechanism of quiescence will be described for different host–pathogen interactions.

### Quiescence in *Colletotrichum*

The hemibiotrophic fungus *Colletotrichum* establishes quiescence in unripe tomatoes by forming melanized appressoria, which penetrate and colonize limited host tissue (Guidarelli *et al*., 2011; Alkan *et al*., 2015) (Fig. 1a). These specialized structures breach the cuticle, facilitating host contact, while infection hyphae undergo developmental reprogramming. Appressorial formation involves the upregulation of 10 450 genes, including those responsible for melanization and glycerol accumulation – key processes that generate turgor pressure to overcome cellular barriers (de Jong *et al*., 1997).

This developmental transition is regulated by critical signaling pathways, such as the cAMP (Lee & Dean, 1993; Shnaiderman *et al*., 2013) and MAPK cascades (Takano *et al*., 2000), which control melanin biosynthesis, whereas yeast Hog1 homologs drive glycerol production. Following penetration, dendritic‐like structures (DLS) form beneath the cuticle (2–5 d post inoculation), though their function remains poorly understood (Cruickshank, 1995), suggesting a possible fruit immune response where one to two fruit cells will die (Alkan *et al*., 2015) (Fig. 1b).

During long quiescence of the germinated appressoria, the fungus exhibits reduced transcriptional activity, expressing only 7903 genes, far fewer than during active necrotrophic infection. Among these, 178 are quiescence‐specific, including key cell cycle regulators such as Cgl‐*Pps1* and *Pas1 cyclin* (Santra *et al*., 2009). Additionally, a large repertoire of candidate‐secreted effectors containing LysM domains are transcriptionally activated. Notably, ChELP1 and ChELP2 bind chitin and chitin oligomers, suppressing chitin‐triggered immune responses in Arabidopsis by inhibiting MAPK activation (Takahara *et al*., 2021). Silencing these genes impairs both immune suppression and appressorium function, confirming their essential role in early infection. These secreted effectors (e.g. ChELP2) may interact with the host PRRs, potentially contributing to the modulation of quiescence (Dodds & Rathjen, 2010). However, it remains unclear whether and how the host ripening differentially regulates fungal development to overcome the induced host response observed during quiescence.

Epigenetic regulation may also contribute to quiescence, as suggested by the presence of histone modifiers (Cgl‐Sin3p, *Cgl‐Hmt*) and chromatin remodelers. Intriguingly, Cgl‐*Med21* (an RNA polymerase II mediator subunit) transcript increases 100‐fold, indicating transcriptional priming for subsequent necrotrophic growth. Furthermore, ammonia‐producing enzymes – such as glutamate dehydrogenase and nitrilase – are activated, implying early ambient alkalinization, a process linked to necrotrophy (Miyara *et al*., 2010; Alkan *et al*., 2013). Mutants in Cg*gdh* or Cg*pacC* exhibit reduced alkalinization during quiescence (Miyara *et al*., 2008; Alkan *et al*., 2013), suggesting that ammonia secretion is critical for host cell death and DLS formation.

At the same time, the tomato responds to the quiescent appressoria by upregulating 2325 transcripts, likely triggered by fungal effectors or cuticle degradation. These include genes for cell recognition, cutin biosynthesis, and ethylene (ET) signaling – unusually active in green fruit – alongside defense genes (*PR‐10*), implying ET‐mediated defenses. Jasmonic acid (JA), ET, and abscisic acid (ABA) pathways are activated, with JA/ET synergizing defenses and ABA antagonizing them (Maor *et al*., 1998). ABA may induce epidermal callose deposition (Qutob *et al*., 2006), while upregulated glycoalkaloids (e.g. tomatine) create an antifungal environment that contributes to quiescence. Tomatine decline during ripening (Itkin *et al*., 2011) mirrors avocado, where reduced antifungals permit activation of quiescence (Prusky, 1996). In summary, quiescence of *Colletotrichum* in mature green tomato arises from host antifungals suppressing pathogenicity, while the fungus accumulates ammonia to kill cells and sustain quiescence. The activation of histone‐modifying enzymes and chromatin‐remodeling complexes functions as a central transcriptional regulatory mechanism, dynamically modifying chromatin structure and modulating RNA polymerase II pausing (Petty & Pillus, 2013). *This coordinated activity suggests that the pathogen programs the transcriptional activation of fungal genes during these early quiescent stages*. Host JA/ET responses restrict growth, maintaining equilibrium until ripening shifts the balance.

### Quiescence in *Botrytis*

*Botrytis* preferentially initiates quiescent colonization in flower tissue that supplies nutrients – such as strawberry stigmatic fluid, cucumber stigmatic exudates, or freshly wounded stems – where appressoria‐like formation is not needed (Fig. 2a). Analysis of the early infection stages of *Botrytis* showed a transcriptional induction of the effector gene *BcLysM1* occurring in both multicellular and unicellular appressoria‐like, as well as during the early infection phase on Arabidopsis leaves (Crumière *et al*., 2025). This effector suppresses chitin‐triggered plant immunity, including the associated reactive oxygen species (ROS) burst in Arabidopsis during infection. Deletion of BcLysM1 leads to delayed infection initiation and reduced pathogenicity on leaves. This raises the question of whether the suppression of the chitin‐induced ROS burst also occurs during host fruit quiescence. Unfortunately, no report described the expression of these effectors during quiescence. In contrast to further confirming the complexity of the effectors in quiescence, it was reported in *Penicillium expansum* (a pathogen that infects exclusively through wounds) that *LysM* knockout strains showed enhanced virulence (Chen *et al*., 2020). A recent study further confirmed this pattern in *Penicillium digitatum*, which carries a Cell Death‐Inducing Effector 1 (Pd*CDIE1*). This effector triggers ROS‐dependent plant cell death, and being evolutionarily conserved, it plays a major role in the virulence of this wound pathogen (Lin *et al*., 2025).

Additional research directions indicated that *B. cinerea* deploys sRNAs and effector proteins to suppress premature host cell death and immune responses, facilitating host colonization before the necrotrophic phase (Weiberg *et al*., 2013; Veloso & van Kan, 2018). These effectors may help establish conditions conducive to quiescence within colonized cells and a crosstalk between autophagy and apoptosis mechanisms that balance the outcome of cell death (Shlezinger *et al*., 2011; Bi *et al*., 2023) (Fig. 2b). The identification of fungal antiapoptotic mechanisms (Shlezinger *et al*., 2011) indicates a potential suppression of host cell death, resembling a transient biotrophic stage where it is still uncertain whether host cell death precedes or follows invasion (Bi *et al*., 2023). Under these conditions, the fungus may successfully suppress the autophagic response enough to keep host cells alive (Shlezinger *et al*., 2011), and in this way, the fungus gains time to grow within the host tissue, accumulating biomass and enzymatic potential (Veloso & van Kan, 2018) at early stages of quiescence. Once the host fruit has undergone maturity‐related changes and the penetrating fungi have reached a sufficient critical mass (Fig. 2), the fungus produces compounds that trigger apoptosis (Minina *et al*., 2014). This mechanism likely explains *Botrytis* quiescence in fruits, where the pathogen initially may preserve host cell viability for a short period before switching to necrotrophic localization. This may explain that, unlike in leaves – where *Botrytis* secretes cell death‐inducing factors, leading to visible necrotic lesions – quiescent fruit infections exhibit no apparent cell death symptoms.

However, few localized cell deaths at infection sites may prime subsequent lesion development (Shlezinger *et al*., 2011; Sharon & Shlezinger, 2013; Leisen *et al*., 2022). During quiescence, the fungus interacts with host cell membranes postgermination, deploying secretory proteins (Zhu *et al*., 2023), disrupting ROS metabolism, and hijacking host RNA interference systems (Nie *et al*., 2025). Early production of cell wall‐degrading enzymes, such as endopolygalacturonase (*BcPG1*), mediates initial host contact before quiescence (Ji *et al*., 2023). *BcPG1* acts dually as a virulence factor and a microbe‐associated molecular pattern (MAMP), recognized by Arabidopsis RLP42/RBPG1 (RESPONSIVENESS TO BOTRYTIS POLYGALACTURONASES1, a leucine‐rich repeat receptor) and tomato *SlFERL* (a plasma membrane kinase for FERONIA‐like). These interactions activate MAPK signaling, inducing immune responses that may enforce quiescence (Poinssot *et al*., 2003; Zhu *et al*., 2017; Zhang & Zhang, 2022; Ji *et al*., 2023). Alternatively, quiescence may involve the receptor‐like cytoplasmic kinase *TRK1 (TPK1b Related Kinase1*), which complexes with tomato LysM Receptor Kinase to regulate chitin‐induced resistance, ROS accumulation, and JA‐dependent immunity via *SlMYC2* – though its role in ripening fruit remains unclear. Other kinases, such as *TPK1b* (*TOMATO PROTEIN KINASE 1b*), further modulate host resistance; their expression, regulated by the transcription factor *SlWRKY3*, can paradoxically enhance or suppress susceptibility to *B. cinerea* (AbuQamar *et al*., 2008), suggesting that these might be the first initial interactions leading to quiescence. Interestingly, these interactions have not been tested in the different fruit tissues where *Botrytis* remains quiescent.

*Botrytis* quiescence may also be associated with Nonexpressor of Pathogenesis‐Related Gene 1 (NPR1), a central regulator of plant defense. NPR1 acts as a salicylic acid (SA) receptor and fine‐tunes ET‐mediated resistance against necrotrophs (Backer *et al*., 2019; R. Li *et al*., 2020). In ripening tomato fruit, SlNPR1 knockout reduced *B. cinerea* infection, leading to smaller lesions, higher defense enzyme activity, and upregulation of defense‐related genes (R. Li *et al*., 2020). Furthermore, SlNPR1 suppression maintained ROS homeostasis by increasing peroxidase (POD), superoxide dismutase (SOD), and glutathione S‐transferase (GST) activity while decreasing catalase (CAT) activity. It also triggered phenylpropanoid biosynthesis and other metabolic pathways, boosting resistance to *B. cinerea*. In addition, NPR1 may induce SA that antagonizes JA, needed by necrotrophs to induce resistance. This inhibition of JA signaling by SA is the result of *B. cinerea* secretion of an exopolysaccharide, allowing the activation of fungal colonization of tomato (El Oirdi *et al*., 2011). Collectively, these findings indicate that SlNPR1 downregulation enhances tomato fruit defenses by modulation of several pathways that could contribute to the early fruit quiescence in unripe fruits.

A detailed transcriptomic analysis of strawberry fruit revealed key differences in gene expression during *Botrytis* quiescence (Petrasch *et al*., 2019), with slower development in flowers and white fruit compared to red, more mature fruit. RNA‐seq at 24 h post inoculation identified 2141 differentially expressed genes (12.5% of total expressed genes), including 60 membrane‐localized receptor‐like kinase genes – enriched in white fruit – that may mediate cell wall–plasma membrane communication. While *B. cinerea* typically activates hypersensitivity‐related (HR) genes to facilitate infection (Govrin *et al*., 2006), its role in early stages of quiescence remains unclear, particularly whether the fungus exploits HR to induce localized cell death. Phytohormone‐related genes (except SA) were differentially regulated post‐inoculation. ET and JA biosynthesis genes (ACC‐oxidase, AOC, OPDA‐reductase, lipoxygenase) were upregulated in both fruit stages, whereas ABA responses were more pronounced in white fruit. Notably, ABA deficiency enhances resistance in tomato and Arabidopsis by altering ROS and cell wall rigidity (Curvers *et al*., 2010). Defense‐related genes, including 28 putative PR proteins, were predominantly altered in white fruit, with marked upregulation of chitinases and differential induction of β‐1,3‐glucanases in white vs red response. PR proteins binding sterols and flavonoids further suggested an earlier, stronger defense response in unripe fruit.

Additionally, cell wall reinforcement through hemicellulose, cellulose, and lignin – may contribute to quiescence of *Botrytis* in grapevine flowers (Haile *et al*., 2017). Studies in grape tissues showed germinated conidia infect inflorescences during bloom, with defense compounds such as resveratrol accumulating in calyptras and receptacles post‐inoculation (Keller *et al*., 2003). Transcriptomic analysis indicated fungal cell wall remodeling – likely to evade host chitinases – during quiescence, alongside sustained metabolic activity (Haile *et al*., 2017). Berries differentially expressed PR proteins and genes involved in monolignol, flavonoid, and stilbenoid biosynthesis, while fungal virulence genes remained suppressed until ripening triggered full activation, potentially facilitated by host cell wall disassembly. These findings suggest that the defense responses in grapevine flowers restrict fungal invasion into underlaying tissues, forcing the fungus into quiescence until conditions favor renewed pathogenic growth. However, the necrotrophic fungus ultimately exits quiescence by deploying a potent cocktail of cell wall‐degrading enzymes, phytotoxic proteins, and secondary metabolites, generating invasive pressure to breach plant cells.

### Quiescence in *Monilinia*

Functional analyses of *Monilinia laxa* in immature and mature peach fruit revealed distinct stage‐dependent strategies (Vilanova *et al*., 2021). During quiescence in immature fruit, *M. laxa* was unable to utilize carbohydrate‐active enzymes (CAZymes) for penetration and struggled against tightly regulated host hormone responses and oxidative bursts, which delayed fungal development over time. By contrast, upon activation in mature fruit, the pathogen relied more on proteolytic effectors than CAZymes and invested early in filamentous growth. Hormone profiling showed that JA likely supported defense in mature fruit, while high ET activity may have increased susceptibility by accelerating ripening. Genes involved in secondary metabolite biosynthesis were more enriched in resistant immature tissue than in susceptible mature fruit, suggesting either host‐produced terpenoids inhibited infection in immature fruit or *M. laxa‐infected* fruits suppressed their biosynthesis in mature tissue (Balsells‐Llauradó *et al*., 2020, 2023). Phenylpropanoid metabolism was strongly induced in both stages, though its role differed: in immature fruit, it likely reinforced cell walls via lignin deposition, whereas in mature fruit, it may have countered fungal ROS detoxification.

Collectively, these findings suggest that fruit immunity during quiescence is characterized by a rapid and synergistic interaction between PTI‐ and ETI‐like processes, which are differentially regulated across host species and developmental stages, including fruit maturity and ripening. This coordinated regulation enhances disease resistance and initiates a cascade of downstream defense responses.

### Quiescence in pathogens that penetrate through wounds

Wound infection of unripe fruit hosts is also a source of quiescent infections for many pathogens. The tomato–*Botrytis* system provides the best‐studied model for comparing host response mechanisms during quiescence of wound pathogens in unripe fruits. Transcriptional profiling of mature‐green‐wounded (resistant) vs mature‐red‐wounded (susceptible) tomatoes infected with *B. cinerea* revealed common defense genes, including receptor‐like kinases, leucine‐rich repeat proteins, WRKY transcription factors, and ET response factors (ERFs) that integrate SA and ET/JA pathways. These shared components, along with JA biosynthesis genes involved in defense and chitin catabolism, mirror known innate immunity pathways, with genes like *LoxD*, *SBT3*, *CEVI‐1*, and *CHI9* appearing in both mature‐green‐quiescent and mature‐red susceptible responses (Danhash *et al*., 1993; Mayda *et al*., 2000; Yan *et al*., 2013; Meyer *et al*., 2016). While some defense genes (e.g. *WRKY33*, *ERF PTI5*) were uniquely expressed in susceptible fruit, the resistant‐stage profile contained functionally similar genes at lower expression levels, suggesting that the activation of quiescence (susceptibility) arises not from the absence of defense genes but from differential regulation (He *et al*., 2001; Gu *et al*., 2002; Wu *et al*., 2016). Ripening‐associated changes critically influence quiescence activation, particularly through cell wall modifications: pectin‐targeting enzymes drive polysaccharide remodeling and softening, which parallels infection‐induced changes in unripe fruit. This link between virulence and ripening is evidenced by pectate lyase tomato mutants showing both delayed softening and reduced *Botrytis* susceptibility, and by polygalacturonase‐deficient *B. cinerea* strains failing to reactivate from quiescence even in ripe fruit (Silva *et al*., 2021). These findings demonstrate that host‐mediated modulation of ripening processes, especially cell wall degradation, is one of the main factors modulating the quiescence in wounded tissue.

## Quiescence exit and virulence onset

The activation of virulence factors during the quiescent stage is driven by four key factors: (1) increased accessibility of disassembled cell wall substrates due to fruit softening during ripening; (2) weakened inducible host‐defense responses; (3) a decline in preformed antifungal compounds such as polyphenols and phytoalexins; and (4) the activation of global virulence factors that modify host environments, often beginning with host pH modulation.

Fungal infection progresses through three simultaneous stages – biotrophic‐quiescent, transition, and necrotrophic – each characterized by distinct host–pathogen interactions. These stages involve waves of host responses and fungal adaptations that collectively increase host susceptibility while triggering pathogenicity genes encoding secreted effector proteins. During the biotrophic‐quiescent phase, fungi employ effectors and cuticle‐degrading enzymes to establish infection. The transition stage introduces additional effectors, cytotoxic compounds, and pH modulators, while the necrotrophic stage is marked by the secretion of cell wall‐degrading enzymes, proteases, and further pH manipulation (Prusky & Yakoby, 2003). These can be summarized in: (1) increased accessibility of disassembled cell wall substrates due to fruit softening during the climacteric stage; (2) weakening of preformed and inducible host‐defense responses; and (3) the activation of global virulence factors that modify host environments, often beginning with host pH modulation. These dynamic shifts in the fungal transcriptome and secretome underscore the pathogen's adaptability and its ability to hijack host ripening regulation, as evidenced by the premature induction of ET synthesis genes during tomato ripening (Cantu *et al*., 2009; Blanco‐Ulate *et al*., 2013). Such simultaneous changes raise a critical question: are the host's physiological modifications during ripening responsible for triggering fungal activation, or do pathogen‐derived virulence factors play the dominant role in driving disease progression (Alkan *et al*., 2008, 2009, 2012; Cantu *et al*., 2008a,b, 2009; Prusky *et al*., 2013)? Although this question remains difficult to resolve, several studies on fungal quiescence in unharvested fruit suggest that host physiological and biochemical shifts are crucial for maintaining resistance. While postharvest pathogens depend on ripening‐related processes and events for pathogenicity, evidence shows that the fungus can also induce these pathways in unripe fruit, indicating that the pathogen is capable of initiating susceptibility by exploiting endogenous developmental programs. Interestingly, postharvest pathogens can colonize fruit from the non‐ripening mutants *rin* and *Cnr*, but not from the *nor* mutant (Cantu *et al*., 2009), suggesting that some, but not all, ripening pathways contribute to susceptibility. Furthermore, numerous studies report that activation of host resistance mechanisms is often associated with delayed fruit maturation and ripening, thereby reducing the conditions that favor increased susceptibility and disease activation (Prusky & Romanazzi, 2023).

## Conclusion

Postharvest pathogens infect produce at pre‐ and postharvest conditions, entering quiescence until ripening weakens host defenses. Pathogens employ *melanized appressoria*, turgor pressure, and nutrient‐sensing to breach cuticles. Biotrophs persist beneath the cuticle of host cells, whereas necrotrophs like *B. cinerea* employ cell death–inducing factors, enhanced by nutrient‐rich target sites, to facilitate penetration but remain dormant until ripening, activating only under favorable conditions. The question is whether quiescence is a fungal inhibition process or an immune response of the unripe host fruit.

Despite factors that inhibit fungal penetration and influence quiescence conditions, biotrophic pathogens such as *Colletotrichum* sense their environment and reprogram their activity through chromatin regulation. By contrast, necrotrophs, such as *Botrytis* and *Penicillium*, maintain quiescence in locations tied to nutrient availability, which enables the expression of early key virulence factors that may regulate quiescence activation stages.

Host responses during quiescence include genes for cell recognition, and ET signaling – usually active in green fruit – alongside defense genes (e.g. *PR‐10*), with JA/ET synergizing defenses and ABA inducing epidermal callose deposition, and upregulated antifungal compounds that all together prevent the activation of quiescence. *Colletotrichum*'s biotrophic hyphae secrete effectors (e.g. ChELP2) interacting with host PRRs, probably contributing to the modulation of quiescence. In necrotrophic pathogens, NPR1, a central regulator of plant defense, acts as an SA receptor and fine‐tunes JA‐ and ET‐mediated resistance against necrotrophs by upregulating defense‐related genes as PR proteins and β‐1,3‐glucanases. Across different pathotypes, the onset and progression of fruit ripening serve as the primary trigger for pathogens to shift from host immune recognition to an active necrotrophic lifestyle. However, the loss of quiescence in ripening fruit appears to result not from distinct response mechanisms in susceptible fruits but from a diminished expression of the same defense‐related pathways.

Emergence from quiescence is attributed to: (1) greater access to cell wall substrates from fruit softening; (2) weakened host defenses; (3) reduced antifungal compounds; and (4) activation of virulence factors modifying the host. Inhibition of fungal growth during the fruit's immune response remains strongly dependent on host metabolism. A deeper understanding of the mechanisms underlying fruit immunity during quiescence is therefore critical to preventing resurgence, which is a major cause of postharvest diseases and losses reaching up to 60% of harvested crops.

## Competing interests

None declared.

## Author contributions

DBP conceptualized the focus of the manuscript, conducted the database search, interpreted the results, wrote the original draft of the manuscript, and supervised and reviewed all the different versions of the manuscript. TC conducted the database search and interpreted the results on part of the research material. BL revised the original draft and other versions of the manuscript. ST revised the final version. YC designed the figures of the review and revised the last version of the manuscript. All authors approved the final version for submission.

## Disclaimer

The New Phytologist Foundation remains neutral with regard to jurisdictional claims in maps and in any institutional affiliations.
